# Retinal Identification Based on an Improved Circular Gabor Filter and Scale Invariant Feature Transform

**DOI:** 10.3390/s130709248

**Published:** 2013-07-18

**Authors:** Xianjing Meng, Yilong Yin, Gongping Yang, Xiaoming Xi

**Affiliations:** School of Computer Science and Technology, Shandong University, Jinan 250101, China; E-Mails: rongmengyuan@gmail.com (X.M.); gpyang@sdu.edu.cn (G.Y.); fyzq10@126.com (X.X.)

**Keywords:** retinal identification, scale invariant feature transform, improved circular gabor transform, iterated spatial anisotropic smooth

## Abstract

Retinal identification based on retinal vasculatures in the retina provides the most secure and accurate means of authentication among biometrics and has primarily been used in combination with access control systems at high security facilities. Recently, there has been much interest in retina identification. As digital retina images always suffer from deformations, the Scale Invariant Feature Transform (SIFT), which is known for its distinctiveness and invariance for scale and rotation, has been introduced to retinal based identification. However, some shortcomings like the difficulty of feature extraction and mismatching exist in SIFT-based identification. To solve these problems, a novel preprocessing method based on the Improved Circular Gabor Transform (ICGF) is proposed. After further processing by the iterated spatial anisotropic smooth method, the number of uninformative SIFT keypoints is decreased dramatically. Tested on the VARIA and eight simulated retina databases combining rotation and scaling, the developed method presents promising results and shows robustness to rotations and scale changes.

## 1. Introduction

Biometric features are unique identifying characteristics of an individual which are more convenient and secure than traditional authentication methods. In traditional authentication systems, identification is based on possessions or knowledge, but when biometrics is used instead, loss of access cards or forgetting passwords can be avoided. Biometrics or biometric authentication [1,2], refers to automated methods of recognizing a person using behavioral or physiological features, such as faces [3], gaits [4], fingerprints [5,6], irises [7], veins [8], *etc.* Among these features, the retina may provide a higher level of security due to its inherent robustness against imposters. Simon and Goldstein [9] discovered already back in 1935 that every eye has its own unique vasculature pattern, even among identical twins. The vasculature of the retina is claimed to be the most secure biometrics, because it is not easy to change or replicate [10]. Although vasculature is thought to be permanent, it still has some limitations. For example, injuries or diseases may alter the vascular features [11] and the public may perceive retinal scanning to be a health threat and time consuming. To date, retinal recognition has primarily been used in combination with access control systems at high security facilities [12,13], such as military installations, nuclear facilities, and laboratories.

Because deformations may exist in retinal images, the SIFT [14] feature is an intuitively good candidate for identification. In this paper, a new retinal identification method based on SIFT and ICGF is presented. SIFT is a novel and promising method for retinal recognition to deal with deformations like rotation, scaling and affine transformation. Moreover, SIFT is robust to pathological lesions because lesions can also serve as salient features, while with the landmark based identification methods, both vasculature lesions and non-vasculature lesions must be taken seriously for they can seriously affect vessel segmentation and subsequent processing. However, the retinal images always suffer from low gray level contrast and dynamic ranges [15] which can affects keypoint extraction and matching and lead to poor performance in SIFT-based identification. In this work, a new approach based on ICGF is proposed for enhancing the details of retinal images, and the background is uniformed simultaneously. Our method is motivated by the seminal work of Li *et al.* [16] which is based on the observation that intensities in a relatively small region are separable, despite the inseparability of the intensities in the whole image. We first define an ICGF template for image intensities in the neighborhood around a pixel, then after being convolved by the template, and additive bias field in obtained from the original image. The image contrast is greatly enhanced after eliminating the bias field from the original image with the unrecognizable capillary vessels being clarified. The generally accepted assumption on the bias field in that it is slowly varying, and it is necessary to preserve the slowly varying property of the computed bias field. The Circular Gabor Filter (CGF) [17] has promisingly good performance in the computation of additive bias fields for its property of anisotropy in scaling and directionality, and therefore is a good candidate for computing additive bias fields.

The details of the retinal image are greatly enhanced after being processed by the ICGF, together with the noises in the image, which increases the number of keypoints redundantly. The iterated spatial anisotropic smooth method [18] is introduced due to its efficient noise reduction ability in homogeneous regions and small structures of retinal vessel are enhanced. Thus feature points can be represented well and some fake feature points are excluded.

To verify the effectiveness of our method, we tested our method on the VARIA [19] retinal database, which is mainly used for recognition. To our best of knowledge, the VARIA database is the only available public retinal database mainly for recognition, as other public retinal databases, such as DRIVE [20] and STARE [21], are built for vasculature segmentation with benchmarks, but there is no class labels for the retinal images. Most of the existing retinal recognition works are tested on images from limited self-built databases or simulated images from these three public databases. To further test the robustness to rotations and scale changes of our approach, we built eight simulated databases from VARIA with different rotation angles and scale changes, respectively.

The rest of the paper is organized as follows: Section 2 reviews the related works. Section 3 introduces the proposed method, followed by a detailed description. Section 4 reports our experimental results. Finally, Section 5 concludes the paper.

## 2. Related Works

Many retina identification methods have been exploited. The first identification system using a commercial retina scanner called EyeDentification 7.5 was proposed by EyeDentify Company in 1976 [22]. Since then, we have witnessed a substantial progress in retinal identification. With a rapid increase of security demands, robust and effective retinal identification has become increasingly important. Many of the existing retinal identification methods are based on extracted vasculatures. However, these approaches may suffer from the following drawbacks: (1) the extracted vasculature information is usually incomplete due to the convergence of multiple and various bent vessels; (2) injuries or diseases can alter the vascular features and lead to improper segmentation; (3) visible micro vascular cannot be extracted efficiently. To address these problems, the minutiae-based retinal identification system (MBRIS) [23–25] was proposed and in recent years it has been considered to be ideal and robust. In a typical MBRIS, a set of landmarks (bifurcations [26] and crossovers of the retinal vessel tree) are extracted and used as feature points. However, due to improper segmentation and unexpected rotation, the matching results returned by these methods may not be effectively accurate.

In addition, the angular partition based method [27–29] has also been used in retinal identification. In [27] the authors claimed that preprocessing based on blood vessel extraction increases the computational time, and proposed a new feature extraction method without any preprocessing phase based on angular partitioning of the spectrum. Another approach that uses angular partitioning for identifying retinal images claimed that the identification task in their approach is invariant from the most of the common affine transformations and is suitable for real time applications [28]. The study of Amiri *et al.* [29] suggested a new human identification system based on features obtained from retinal images using angular and radial partitioning of the images. A different algorithm is proposed for the extraction of the retinal characteristic features based on image analysis and image statistics in [30]. In this study, a Hue, Saturation, and Intensity (HSI) color model is used and the features are extracted from the plane of fundus images captured by a fundus camera. These features are used for either the verification or identification process. The study tested the algorithm on sixty fundus images and received a success rate of 94.5%.

SIFT features have been previously used in adaptive optics retinal image registration [31]. In [31], Li *et al.* use the SIFT algorithm to automatically abstract corner points and match the points in two frames, moreover the motion of the retina is corrected. In [32], Li *et al.* tracked features of the adaptive optics confocal scanning laser retinal image using the KLT-SIFT algorithm. And according to the tracked points, complex distortions of the frames have been removed. As SIFT is known for its distinctiveness and invariance to scale and rotation, it is widely used in object recognition. In this paper, we introduce the SIFT feature to retinal identification. Retinal images acquired with a fundus camera often suffer from low gray level contrast and dynamic range, which can seriously affect keypoint extraction and cause mismatching. To improve the quality of retinal images, Feng *et al.* [15] proposed an enhancing method based on Contourlet transform. Their method provides an effective and promising approach for retinal image enhancement, however, can′t effectively ameliorate the performance of subsequent identification based on SIFT. In [33], Marín *et al.* proposed a background homogenizing method as a preprocessing step for vasculature segmentation. In their method, the estimation of the background is the result of a filtering operation with a large arithmetic mean kernel. In [34], Foracchia *et al.* proposed a luminosity and contrast normalization method in retinal images. Their method is based on the estimation of the luminosity and contrast variability in the background part of the image and the subsequent compensation of this variability in the whole image. Both of these methods can facilitate automatic fundus image analysis, but lack the ability to enhance the detail of retinal images sufficiently.

A summary of retinal identification approaches with typical references is provided in Table 1, which should help researchers conduct comparisons with the existing works and evaluate their own work. From Table 1, we can figure out that most of the existing works are conducted on self-built databases with a limited number of retina images, except for the method proposed in [25]. In Section 4, we also conduct an experiment comparing our work with the seminal work in [25], which is one of the best and most reliable existing methods. In their work, a new vessel-directional feature descriptor called the principal bifurcation orientation (PBO) is proposed and achieved improved robustness. We referenced this work as the PBO based method in Section 4.

## 3. System Architecture

**Figure 1.**
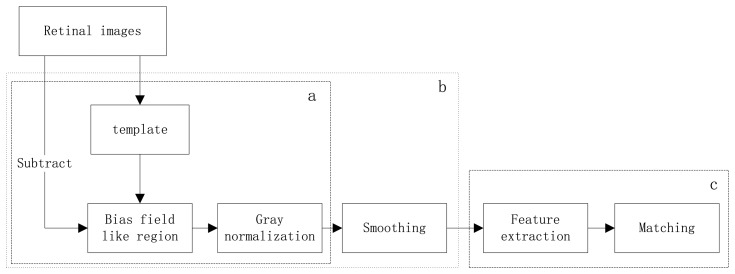
Flowchart of the proposed method. (**a**) Background uniformation; (**b**) preprocessing; (**c**) SIFT based recognition.

The herein proposed identification method consists of two main phases: preprocessing and SIFT-based recognition, as is systematically described by the functional block diagram in Figure 1. These phases are further subdivided into several steps, as follows:

Preprocessing: (1) Background uniformation. This step normalizes the nonuniformly distributed background by removing the bias field like region, which is acquired by convolve the original image with the ICGF template, from the original retinal images. Then the intensity of the new image is normalized to values from 0 to 255. (2) Smoothing. Reduce the noise in homogeneous regions using the iterated spatial anisotropic smooth method. What's more, small structures of retinal vessels are enhanced.

Recognition: (1) Feature extraction. Stable keypoints are extracted by using the SIFT algorithm, the keypoints can characterize the uniqueness of each class. (2) Matching. Find the number of matched pairs in two retinal images. Each step is detailed and illustrated in the following sections.

### 3.1. Preprocessing

#### 3.1.1. Improved Circular Gabor Filter

Circular Gabor Filters (CGF) were introduced and applied into invariant texture segmentation by Zhang *et al.* [17]. It is the modification of traditional Gabor filters. Traditional Gabor filters are well known as orientation detectors, but in the application of extracting orientation invariant features, their main advantages become vital. Circular Gabor filters are suitable for extracting rotation invariant features because there is no concept of direction in them. The CGF [39] is defined as follows:

(1) $$G(x,y)=g(x,y)\exp(2\pi jF\sqrt{x^{2}+y^{2}})$$

where *F* is the central frequency of a circular Gabor filter, and *g*(*x*,*y*) is a 2-D Gaussian envelope assumed to be isotropic, which is defined as:

(2) $$g(x,y)=(\frac{1}{2\pi \sigma^{2}})\exp(-\frac{x^{2}+y^{2}}{2\sigma^{2}})$$

**Figure 2.**
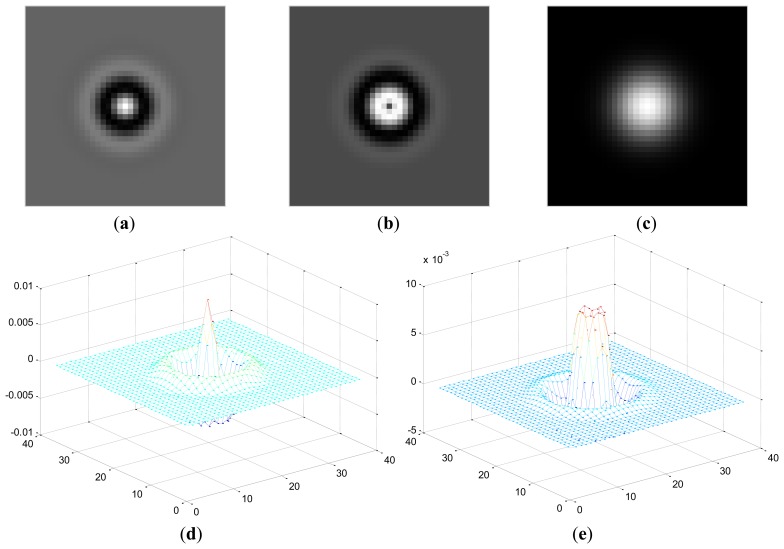
(**a**) and (**b**) are the 2-D views of the real and imaginary part of CGF(*σ* = 4, *F* = 32/256); (**c**) is the magnitude image; (**d**) and (**e**) are the 3-D views of (a) and (b), respectively.

**Figure 3.**
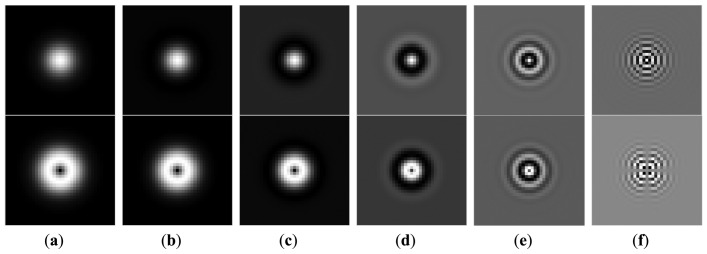
A bank of CGF with *σ* = 4, *F* = {4,8,16,32,64,128}/256. The first row is the real parts and the second row is the imaginary part.

The Fourier representation of the circular Gabor filter is as follows:

(3) $$F(u,v)=\frac{\sqrt{2\pi }}{2}\alpha \exp(-\frac{{\left(\sqrt{u^{2}+v^{2}}-F\right)}^{2}}{2\alpha^{2}})$$

where $\alpha =\frac{1}{2\pi \sigma }$. Frequency domain can better display the properties of a circular Gabor filter. 2-D and 3-D view of circular Gabor filters looks like a ring in frequency domain while it is similar to rippling water waves in spatial domain. Figure 2 shows an example of 2-D and 3-D view of circular Gabor filters in spatial domain. For a texture image of size *N* × *N*, the frequency *F* is often selected as follows(normalized by *N*): $\sqrt{2}\{1,2,4,8,16,\ldots ,\overset{N}{\;}/\underset{4}{\;})/N$. Here, we choose a bank of CGF with different central frequency *F* = {4,8,16,32,64,128}/256, which is shown in Figure 3.

In the preprocessing of the retina image, some adjustments are made. In the original circular Gabor filter, the texture property of each pixel in the image is provided by the projection of the textured surface *I*(*x*,*y*) onto a complex Gabor wavelets [17]. This can be defined as:

(4) P=∬I(x,y)G(x,y)dxdy

In our method, we define:

(5) $$P=\iint I(x,y)(Z_{r}(x,y)-Z_{i}(x,y)i)\text{dxdy}$$

**Figure 4.**
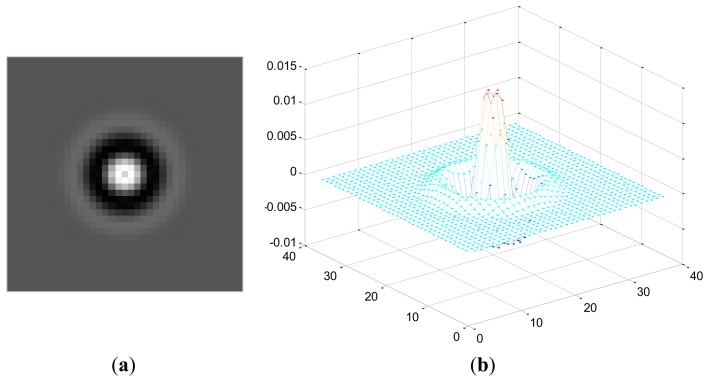
2-D and 3-D views of the new template *Z_r_*(*x*,*y*) - *Z_i_*(*x*,*y*)*i*.

**Figure 5.**
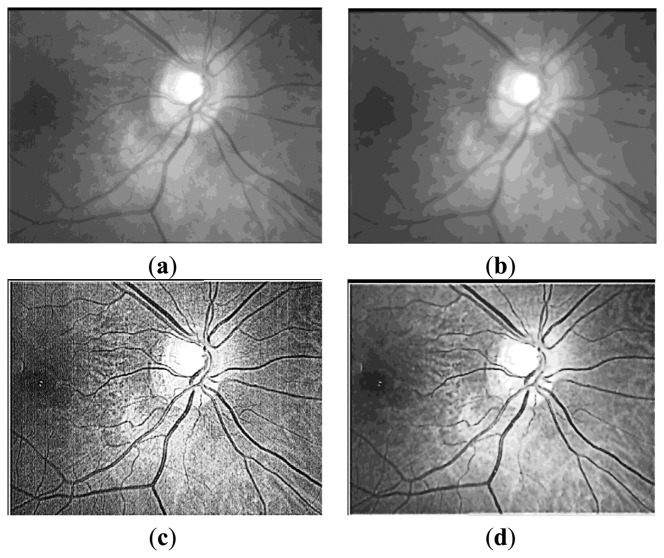
(**a**) The original retina image; (**b**) the bias like image which is (a) processed by the ICGF; (**c**) (a) subtract (b); (**d**) Result image of preprocessing which is (c) after nonlinear anisotropic diffusion.

Here, *Z_r_*(*x*,*y*) represents the coefficient matrix of the real part of CGF and *Z_i_*(*x*,*y*) stands for the imaginary part. This operation means the retina images is convolved by the new template which is sum of coefficient of both the real and imaginary part of the original CGF, as can be seen in Figure 4. In this paper, we set *F* = 2.1, *σ* = 3.7 for the template. The result *P* after convolution is shown in Figure 5b, It should be noted that for viewing convenience, *P* is normalized to values from 0 to 255. From the image shown in Figure 5b, we can see that *P* is a bias-like image generated from the original retinal image.

The final retinal image is defined as:

(6) $$R^{'}=I-P$$

(7) $$R=\frac{R^{'}-\min(R^{'})}{\max(R^{'})-\min(R^{'})}\times 255$$

Here, *R* is the final retinal image resulted from the proposed ICGF. This operation is simulated as removing the additive bias from the original retinal image. *R*′ is the temporal variable, its values range from [−255, 255] and is normalized to [0, 255] for further processing. *R* is shown in Figure 5c, we can see that the image processed by the ICGF has a stronger gray level contrast than the original retinal image, and has a substantially uniform grayscale distribution.

#### 3.1.2. Iterated Spatial Anisotropic Smooth

The iterated spatial anisotropic smooth method proposed by Gerig *et al.* [18] is based on anisotropic diffusion, which is proposed by Perna and Malik in 1990. Results in [18] demonstrate the efficient noise reduction ability of the spatial anisotropic smooth method in homogeneous regions. In addition, the small structures of retinal vessel are enhanced, as shown in Figure 5d. In this method, Smoothing is formulated as a diffusive process, which is suppressed or stopped at boundaries by selecting locally adaptive diffusion strengths. In any dimension this process can be formulated mathematically as follows:

(8) $$\frac{\partial }{\partial t}u(\bar{x},t)=\text{div}(c(\bar{x},t)\nabla u(\bar{x},t))$$

The diffusion strength is controlled by *c*(*x̄*, *t*). The vector *x̄* represents the spatial coordinates. The variable *t* is the process ordering parameter; in the discrete implementation it is used to enumerate iteration steps. The function *u*(*x̄*, *t*) is taken as image intensity *I*(*x̄*, *t*). The diffusion function *c*(*x̄*, *t*) depends on the magnitude of the gradient of the image intensity. It is a monotonically decreasing function *I*(*x̄*, *t*) = *f*(|∇*I*(*x̄*, *t*)|), which mainly diffuses within regions and does not affect region boundaries at location of high gradients. Two different diffusion functions have also been proposed:

(9) $$c_{1}(\bar{x},t)=\exp\left(-{\left(\frac{\nabla I(\bar{x},t)}{\kappa }\right)}^{2}\right)$$

(10) $$c_{2}(\bar{x},t)=\frac{1}{1+{\left(\frac{\left|\nabla I(\bar{x},t)\right|}{\kappa }\right)}^{1+\alpha }}|\alpha >0$$

### 3.2. SIFT Based Recognition

As the acquired retina image has irregular shadings around the border of the image which may interfere with the keypoints extraction, we simply abandon the border region of the retina image. The segmented region *R*, is captured as follows:

(11) $$R=I(h_{1}:h_{2},w_{1}:w_{2})$$

In our experiment, we set *h*_1_ = 30, *h*_2_ = 530, *w*_1_ = 20, *w*_2_ = 720. 3.2.1. Feature Extraction

#### 3.2.1. Feature Extraction

Scale Invariant Feature Transform (SIFT) approach was proposed by Lowe [14,30] for extracting distinctive invariant features from images. The SIFT algorithm consists of four major stages of computation: (1) Scale-space extrema detection; (2) Unreliable keypoints removal; (3) Orientation assignment; (4) keypoint descriptor. Following is a brief introduction of each stage:

The first stage computes the locations of potential interest points in the image by detecting the maxima and minima of a set of Difference of Gaussian (DoG) images obtained at different scales all over the image. The DoG image *D*(*x*,*y*,*σ*) is the difference of smoothed *L*(*x*,*y*,*σ*) images:

(12) D(x,y,σ)=L(x,y,kσ)−L(x,y,σ)

where *L*(*x*,*y*,*σ*) is obtained from variable scale Gaussian with the input image *I*(*x*,*y*):

(13) $$L(x,y,\sigma )=\frac{1}{2\pi \sigma^{2}}\exp\left(-\frac{x^{2}+y^{2}}{2\sigma^{2}}\right)\times I(x,y)$$

**Figure 6.**
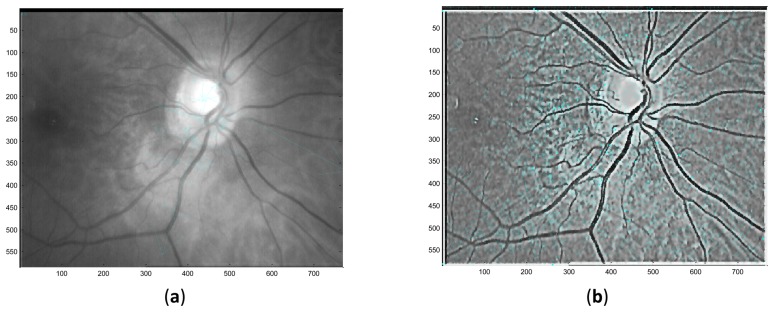
Illustrations of the distributions of keypoints. (**a**) keypoints distribution before enhancement; (**b**) keypoints distribution after enhancement.

Performed in different scales *σ*, numbers of DoG images are obtained. Local extrema are detected from these DoG images by comparing a pixel with its neighbors in 3D space and if it is an extremum, this pixel is selected as a keypoint. In the second stage, the candidate keypoints are refined by discarding points of low contrast. If the referenced value at one candidate keypoint is below a certain threshold which means that this keypoint is of low contrast and the keypoint will be removed. In the third stage, one or more orientations are assigned to each keypoint based on local image gradient directions. In the final stage, a local feature descriptor is computed at each keypoint based on a patch of pixels in its local neighborhood. Figure 6 illustrates the distribution of keypoints in retinal images.

#### 3.2.2. Matching

**Figure 7.**
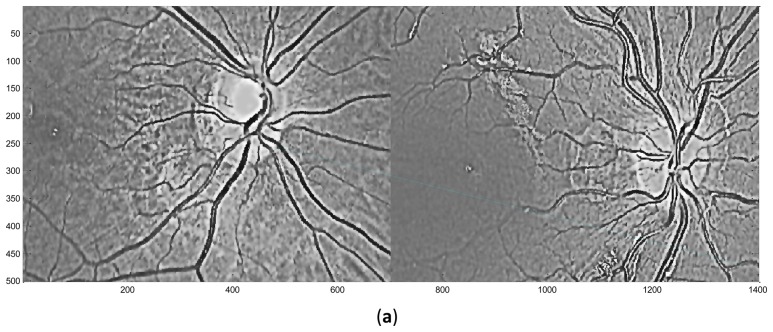

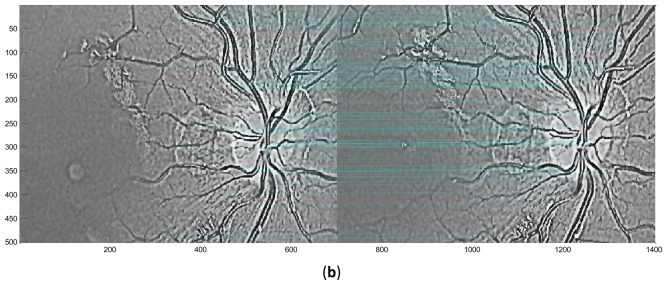
Exemplary matching results. (**a**) Imposter matching with very few (just one) matching pairs; (**b**) Genuine matching, with 142 matches.

In this stage, feature descriptors extracted from two retinal images are matched. Based on the extracted feature descriptors, the number of matching pairs is used to measure the similarity of these two retinal images. Then the suitable threshold *T* (the number of matching pairs) is selected after testing the whole retinal database. The two retinal images will be classified into the same class if the number of matching pairs is bigger than *T*, otherwise, these two retinal images will be classified as different classes. Figure 7 shows the exemplary matching results.

## 4. Experimental Results and Analysis

### 4.1. The Experimental Database

In biometric based authentication systems, the accuracy of implemented algorithms is very important and they must be tested properly. In this paper, the experiments were conducted on two kinds of retinal databases. The primary database is a subset of the VARIA [30] database which is built for retinal recognition systems. This database includes 233 retinal images with a resolution of 768 × 584 from 139 different subjects, 59 of which possess at least two samples. The images have been acquired over a time span of several years with a TopCop NW-100 model non-mydriatic retinal camera. These images are optic disc centered and have a high variability in contrast and illumination, allowing the system to be tested in quite hard conditions and simulating a more realistic environment. The different conditions are also due to the fact that different experts with different illumination configurations on the camera have acquired the images. As a recognition problem, every subject must have more than one sample, so we choose the above mentioned 59 subjects include 153 samples in total. For the secondary database, to test the robustness in rotation and scale invariance, we created eight simulated rotated and scaled databases respectively based on the primary database. The four rotation angles are ±10°, ±20°, ±30° and ±40°. The scale ranges are 1 ± 0.05, 1 ± 0.10, 1 ± 0.15 and 1 ± 0.20.

### 4.2. Experimental Settings

All the experiments are implemented in MATLAB, and performed on a PC with a 2.4 GHz CPU and 2.0 G Byte memory. The experiments are designed to multilaterally evaluate the proposed method: (1) Experiment 1 evaluates the proposed method in the verification mode; (2) In Experiment 2, the effectiveness of every step of the proposed enhancement method is analyzed; (3) To test the robustness and invariance of the proposed method, we conduct Experiment 3 on the secondary database in the verification mode and compared with one of the best previous method. (4) In Experiment 4, the average processing time of proposed method is measured and compared with the PBO method.

### 4.3. Experiment 1

We performed Experiment 1 on the primary database in the verification mode. In this mode, the class of the input retina is known, and each sample is matched with all the other samples from the same subjects and all samples from the other 58 subjects. A successful matching is called intraclass matching or genuine, if the two matching samples are from the same subjects. Otherwise, the unsuccessful matching is called interclass matching or imposter. As mentioned above, we use full matching in intraclass (each sample is matched with all the other samples from the same class) and interclass matching (each sample is matched with all the samples from other 58 subjects). Consequently, there are 155 intraclass matching and 11,473 interclass matching in total. In this paper, the performance of a system is evaluated by the EER (equal error rate) the FAR (false accept rate) at zero FRR (false rejection rate) and FRR at zero FAR. The FAR value for a matching score is stated as the number of imposter comparisons with score higher than this matching score divided by the total number of imposter comparison. The FRR value for a matching score is the number of genuine comparisons with score lower than this score divided by the total number of genuine comparison. The EER is the error rate when the FRR equals the FAR and therefore suited for measuring the performance of biometrics systems because the FRR and FAR are treated equally. On the other hand, the FRR at zero FAR is suited for high security systems, as in those systems, false acceptance errors are much more critical than false rejection errors. On the contrary, the FAR at zero FRR shows the acceptance rate of imposters when more genuine rejected is desired.

The EER of the proposed method is 0. Genuine and imposter matching similarity distribution is shown in Figure 6. As mentioned above, we take the number of matching pairs as the similarity of two retinal images. And the number of matching pairs in a genuine matching could be much larger than in imposter matching. And the similarity distribution could have a large range in one figure. It is the intersection point of the two similarity distributions that should be emphasized. And we conduct this operation to have a more descriptive visualization:

(14) $$\begin{array}{c} S_{g}^{'}=\frac{S_{g}}{2\times \max(\max(S_{g}))}\\ S_{i}^{'}=\frac{S_{i}}{2\times \max(\max(S_{g}))} \end{array}$$

**Figure 8.**
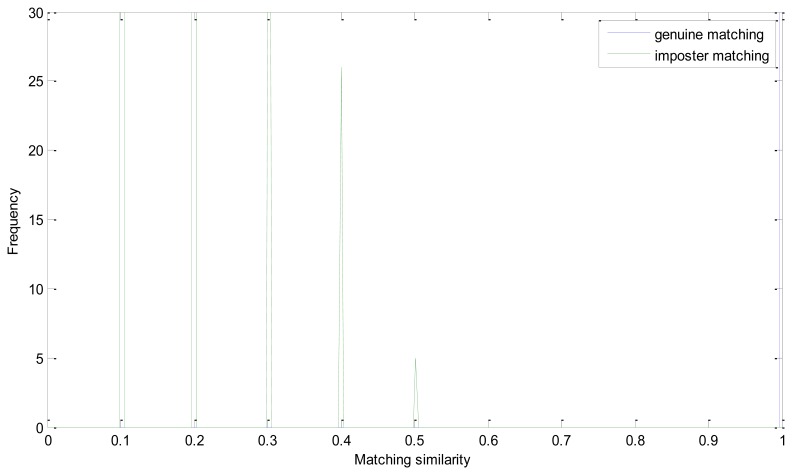
Distribution of matching similarity of the proposed method.

In Equation (14), *S_g_* and *S_i_* represent matrix of the number of matching pairs of the genuine and imposter matching, respectively. Apparently, *S_g_*′ ranges from 0 to 0.5 and *S_i_*′ is mainly between 0.5 and 1. And if the EER is zero, all of the number in *S_i_*′ must be larger than 0.5. From Figure 8, we can see that the similarity of the imposter matching is between 0 and 0.5, and the genuine similarity is larger than 1. These two distributions have no intersection and there is a large margin between the two similarity distributions.

### 4.4. Experiment 2

**Figure 9.**
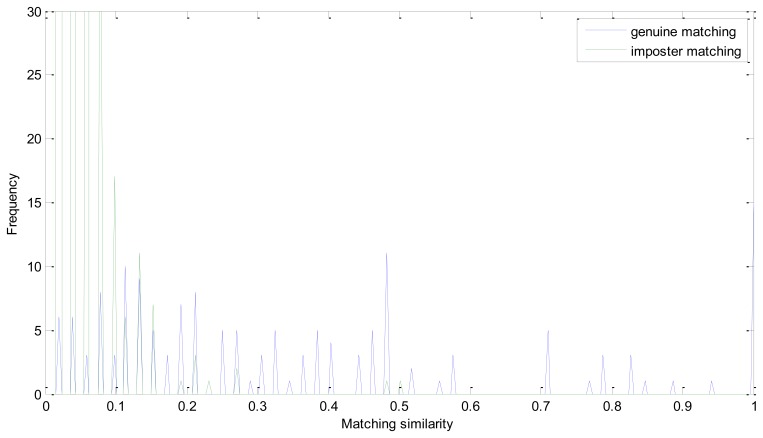
Similarity distribution without enhancement.

**Figure 10.**
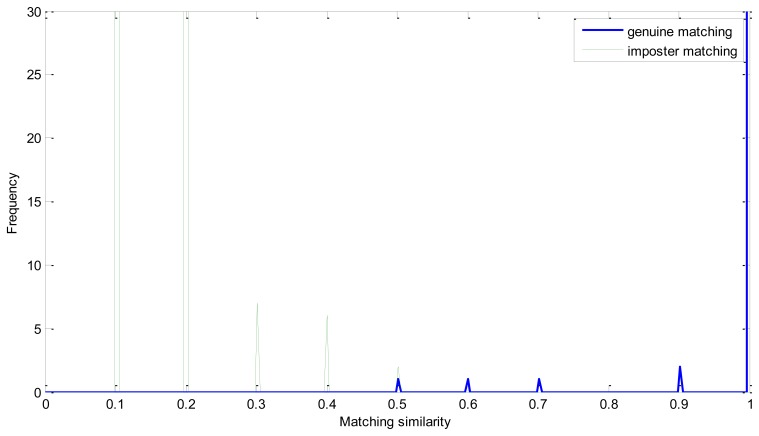
Similarity distribution without smooth.

We analyze the effectiveness of every step of the proposed enhancement method in Experiment 2, *i.e.*, without enhancement, without iterated spatial anisotropic smooth and the proposed method. Figures 9 and 10 show the similarity distribution without enhancement and without smooth, respectively. From the distributions we can see that, when without any enhancement, the imposter matching similarity is ranging from 0 to 1, which intersects with the genuine similarities. After processed by the ICGF, the imposter matching similarities is larger than 0.5, but still has some intersection.

**Figure 11.**
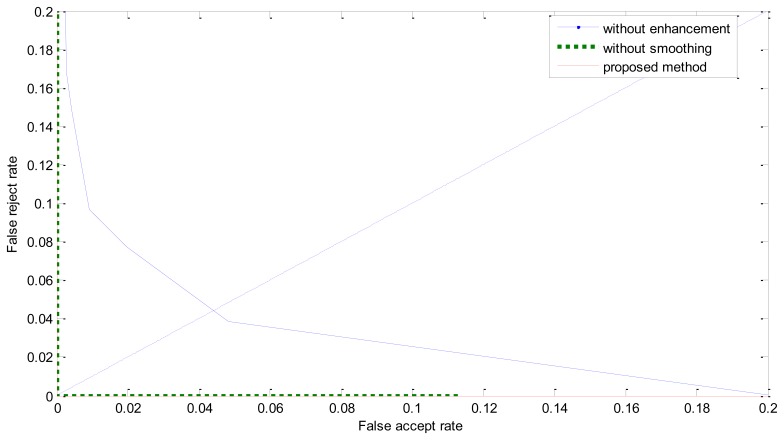
The ROC curve in the verification mode.

The ROC curves are shown in Figure 11. The FRR at zero FAR and FAR at zero FRR values are listed in Table 1. From Figure 11 and Table 2, we can see that the proposed method achieves a much lower EER. This indicates that the enhancement method introduced in this paper is effective. From Figure 8 we can also see that the proposed method has potential robustness for larger databases because there is a large margin between the genuine and imposter matching.

**Figure 12.**
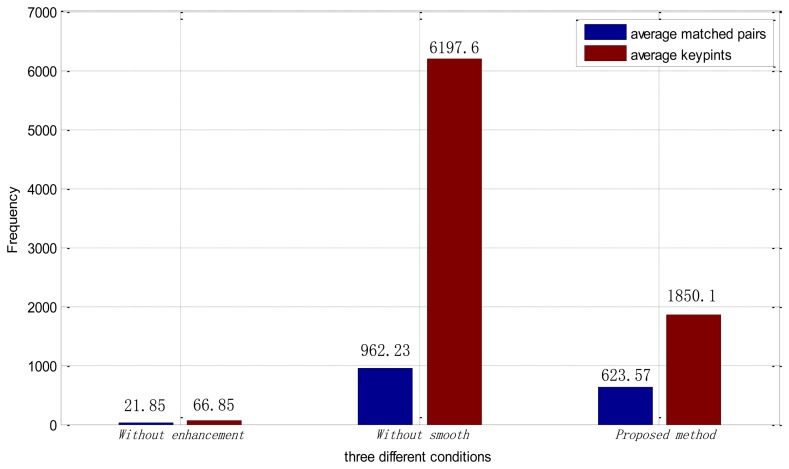
Keypoints analysis.

We also analyzed the average number of keypoints and average number of matching pairs in each method. From Figure 12, we can see that, after being processed by the ICGF, the average number of keypoints finding in each retinal image is about 90 times larger. The number of average matching pairs is 15.5 percent of it. While in the proposed method, the average number of keypoints in each retinal image drops to 1,850.1, and the proportion of the average matched pairs in each image is increased to 33.7 percent. Large numbers of keypoints can lead to a much more time consumption in the feature extraction and matching procedure. The proposed method improves the number of feature points extracted and eliminates the interference information at the same time.

### 4.5. Experiment 3

**Figure 13.**
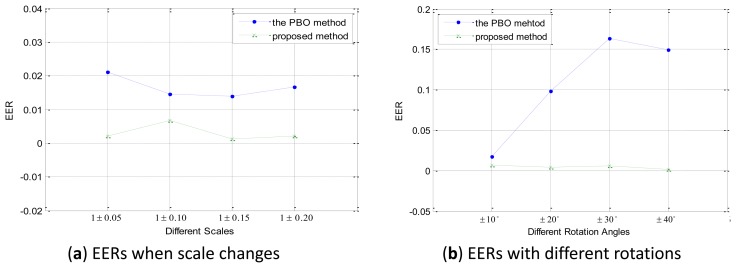
Performance on the secondary databases.

To test the robustness to scale changes and rotations of the proposed method, we conduct Experiment 3 on the secondary database in the verification mode. As mentioned in Section 4.1, each secondary database includes retina images from the primary database and a simulated datasets and every class has at least six samples. We use full matching, and consequently, there are 1,854 intraclass matching and 103,257 interclass matching in total. Figure 13 shows the EERs of the proposed method on each secondary set, respectively. From the figure we can see that, for the proposed method, though the values of EER varies according to rotations or scale changes, all the values of EER is no bigger than 0.0065 which indicates that the proposed method is robust to rotations and scale changes. While due to the lack of robustness of rotations or scale changes, which are common deformations in normal retina images, the performance is of the PBO method changes dramatically.

### 4.6. Experiment 4

In Experiment 4, the average processing times were measured, as shown in Table 3. The experiment is implemented in MATLAB, and conducted on a PC with a 2.4 GHz CPU and 2.0 G Byte memory. The average preprocessing time is 0.28 s when smoothing is eliminated, which is much faster than the proposed method, but the processing time of feature extraction and matching is dramatically increased. From Table 3 we can see that the total processing time of the proposed method is about half of the method without smoothing. The total average processing time of the proposed method is 7.87 s, which is very time-consuming. Comparing to the PBO method which is 2.31 s in total, the SIFT-based method is much slower but both are in seconds. There are several ways to reduce the computation time. The scale of retina images used in our experiment is relatively large and the processing time can decrease relatively according to the scale changes. Furthermore, MATLAB is an integrated development environment, which can also affect the time consumption. In addition, because most of the matching pairs concentrate around the optical disk and vessel network, effective definition of Region of Interest also can help to reduce time consumption. Above all, the iterated spatial anisotropic smooth decreases the uninformative keypoints and have reduced the time consumption of the retinal identification system.

## 5. Conclusion and Future Work

Retinal identification based on retinal vasculatures provides the most secure and accurate authentication means among biometrics. In this paper, we have presented a robust retinal identification method based on SIFT and ICGF. SIFT is invariant to rotations and scale changes. In addition, the SIFT-based identification method can abolish the image segmentation or partition procedures and doesn′t need any comprehension of the image contents.

The method proposed in this paper has proved to be effective for the identification based on retinal images. The results reported in Table 2 show that, SIFT-based identification based on the original retinal images has an undesirable performance. After being processed by the ICGF, the performance is remarkably improved, but still has room for improvements. Further smoothed by the iterated spatial anisotropic smooth method, the EER reaches 0. In addition, from the perspective of the average keypoints analysis, as illustrated in Figure 12, the presented ICGF increased the average number of stable keypoints about 90 times by enhancing the details of the images. After being smoothed, the proportion of average number of matching pairs is greatly increased with noise reduced and small structure enhanced.

When compared with other retinal identification approaches, most of the existing research works have been tested on the VARIA retinal database or self-built databases with a limited number of images, except the PBO method, as listed in Table 1. The performance of the proposed method is comparable to the existing methods, and in some cases, exceeded their results. Further tested on simulated databases, Figure 13 shows that the proposed method has robustness to scaling and rotations, and has a superior performance compared with the PBO method. The proposed identification method based on SIFT features and ICGF can be used on other biometric patterns, such as hand veins and finger veins. In the future, we plan to design new feature extraction methods which can be more robust to image deformations.

## Figures and Tables

**Table 1.** Methods for retinal identification.

**References**	**Method**	**Database**	**Performance**
[12]	vascular segmentation	self-built database: 400 STARE database: 80	recognition rate: 95%
[13]	vascular segmentation	Self-built database: 60 × 5	EER (Equal Error Rate): 0.01
[35]	Blood vessel skeleton	Self-built database: 200	38 false rejection and 0.19% recognition rejection
[23]	Minutiae in optical disk	Self-built database: 152 (4 × 2 and 12 × 12)	Recognition rate: 100%
[36]	Optical disk location C-Means clustering	Self-built database: 108 images from 27 people.	-
[37]	Wavelet energy feature Vessel pattern extraction	Self-built database: 40 × 10	Recognition rate: 100%
[27]	Optical disk location, angular partition Angular and radial	DRIVE database: 40	Recognition rate: 100%
[28]	partitioning based on vascular sketch	Self-built database: 40 × 9	Recognition rate: 98%
[25]	Minutiae features from segmented vasculature.	VARIA database Self-built database: 2,063 images from 380 subjects	EER: 0 EER: 0.0153
[20]	Image analysis and image statistics	Self-built database: 30 × 2	Recognition rate: 94.5
[38]	Landmarks from extracted vessel tree.	VARIA database: training 150, testing 40 × 2	EER: 0

**Table 2.** Verification performance of different method.

**Method**	**EER**	**FAR at Zero FRR**	**FRR at Zero FAR**
Without enhancement	0.0436	0.2003	0.7677
Without smooth	87,161e-05	1.7432e-04	0.0065
Proposed method	0	0	0

**Table 3.** The average preprocessing time.

**Method**	**Preprocessing**	**Feature Extraction**	**Matching**
Without smoothing	0.28 s	3.88 s	9.72 s
Proposed method	2.11 s	2.51 s	3.25 s
The PBO method	1.37 s	0.21 s	0.73 s
